# High-Dose Vitamin C: Preclinical Evidence for Tailoring Treatment in Cancer Patients

**DOI:** 10.3390/cancers13061428

**Published:** 2021-03-20

**Authors:** Manuela Giansanti, Terry Karimi, Isabella Faraoni, Grazia Graziani

**Affiliations:** 1 Department of Systems Medicine, University of Rome Tor Vergata, Via Montpellier 1, 00133 Rome, Italy; manuelagiansanti@libero.it (M.G.); terrykarimi.tk@gmail.com (T.K.); faraoni@med.uniroma2.it (I.F.); 2 Department of Physiology and Pharmacology “V. Erspamer”, Sapienza University of Rome, 00185 Rome, Italy

**Keywords:** vitamin C, ascorbate, TET2, 5-hydroxymethylcytosine, PARP inhibitors, IDH1/2, WT1, hypomethylating agents, ROS, DNA damage

## Abstract

**Simple Summary:**

Vitamin C is an indispensable micronutrient in the human diet due to the multiple functions it carries out in the body. Reports of clinical studies have indicated that, when administered at high dosage by the intravenous route, vitamin C may exert beneficial antitumor effects in patients with advanced stage cancers, including those refractory to previous treatment with chemotherapy. The aim of this article is to provide an overview of the current scientific evidence concerning the different mechanisms of action by which high-dose vitamin C may kill tumor cells. A special focus will be given to those mechanisms that provide the rationale basis for tailoring vitamin C treatment according to specific molecular alterations present in the tumor and for the selection of the most appropriate companion drugs.

**Abstract:**

High-dose vitamin C has been proposed as a potential therapeutic approach for patients with advanced tumors who failed previous treatment with chemotherapy. Due to vitamin C complex pharmacokinetics, only intravenous administration allows reaching sufficiently high plasma concentrations required for most of the antitumor effects observed in preclinical studies (>0.250 mM). Moreover, vitamin C entry into cells is tightly regulated by SVCT and GLUT transporters, and is cell type-dependent. Importantly, besides its well-recognized pro-oxidant effects, vitamin C modulates TET enzymes promoting DNA demethylation and acts as cofactor of HIF hydroxylases, whose activity is required for HIF-1α proteasomal degradation. Furthermore, at pharmacological concentrations lower than those required for its pro-oxidant activity (<1 mM), vitamin C in specific genetic contexts may alter the DNA damage response by increasing 5-hydroxymethylcytosine levels. These more recently described vitamin C mechanisms offer new treatment opportunities for tumors with specific molecular defects (e.g., HIF-1α over-expression or TET2, IDH1/2, and WT1 alterations). Moreover, vitamin C action at DNA levels may provide the rationale basis for combination therapies with PARP inhibitors and hypomethylating agents. This review outlines the pharmacokinetic and pharmacodynamic properties of vitamin C to be taken into account in designing clinical studies that evaluate its potential use as anticancer agent.

## 1. Introduction

Vitamin C is an indispensable micronutrient in the human diet due to the multiple functions it carries out in the body. In fact, humans are one of the few species unable to synthesize vitamin C through the oxidation of glucose due to the lack of gulonolactone (L-) oxidase. The importance of vitamin C in human diet was discovered in the 17th century, when the British Royal Navy surgeon James Lind managed to reduce the onset of scurvy (a disease consequent to a prolonged vitamin C deficiency) by introducing citrus fruits, such as lemons and oranges, into the diet of British navy sailors [1]. It was around the 1930s that Albert Szent-Györgyi (Nobel Prize for Medicine in 1937) and Charles Glen King isolated vitamin C [2]. This vitamin participates as co-factor to the hydroxylation of proline and lysine residues of type 1 collagen; thus, it is essential for the formation of collagen and helps maintaining the integrity of the connective and bone tissues, as well as tooth dentin [3]. When vitamin C intake is below 10 mg/d for long periods (>1 month), failure of wound healing, small hemorrhages, bleeding gums, keratosis pilaris, and other systemic and dermatological conditions occur [4,5,6,7,8].

Starting in the second half of the twentieth century, the pioneering studies of Pauling and Cameron proposed vitamin C as a useful agent for the prevention and treatment of cancer [9,10,11]. In these studies, vitamin C was administered first intravenously (I.V.) and then orally as maintenance therapy. Conversely, Moertel and colleagues, showed no positive effects of orally administered vitamin C in cancer patients [12,13]. The debate on vitamin C antitumor efficacy is still ongoing and a number of studies are attempting to establish its role in the treatment of cancer [14]. In this context, particularly interesting are the results of a high number of in vitro studies showing that vitamin C exerts cytotoxic activity on cancer cells, while it is devoid of toxic effects toward normal cells. A number of issues, including the complexity of the biochemical mechanisms regulated by vitamin C and the different experimental models utilized to evaluate its antitumor activity, make it difficult to draw unequivocal conclusions from the preclinical studies carried out over the years. Furthermore, the clinical studies performed in cancer patients in the last decades have shown vitamin C efficacy only in some cases, but biomarkers for predicting patients’ response have not been identified, yet [11,13,15]. Based on the experimental evidence accumulated so far, this review aims at summarizing the pharmacokinetic and pharmacodynamic properties of vitamin C that should be taken into account when designing clinical trials aimed at evaluating its use as anticancer agent.

## 2. Methods

**Figure 1:**
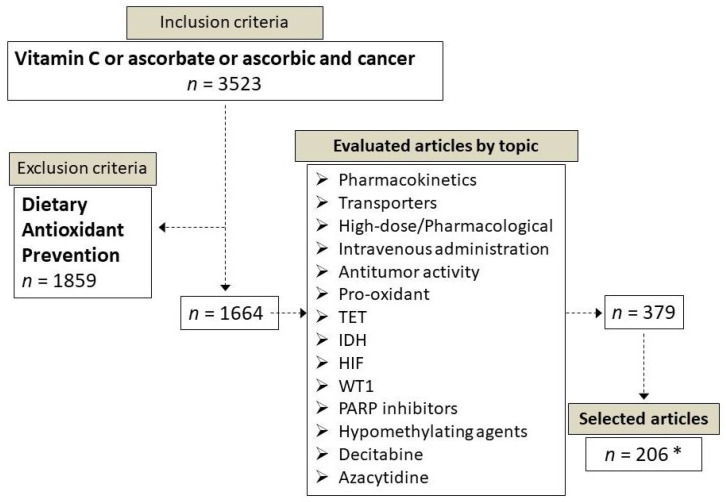
Flow chart of the inclusion and exclusion criteria applied for the selection of the articles cited in this review. * This number includes also articles providing background information that were retrieved by using specific PubMed queries.

Medline was searched via PubMed for relevant, English-written articles published until February 2021. In the preliminary search the following inclusion criteria were applied (in abstract and title): vitamin C or ascorbate or ascorbic and cancer; this search retrieved 3523 articles. By applying dietary, antioxidant, and prevention as exclusion criteria we obtained 1664 articles, among which only 379 were actually relevant to vitamin C used as pharmacological agent and regarded the following topics: pharmacokinetics, transporters, high-dose and pharmacological, intravenous administration, antitumor activity, pro-oxidant, TET, IDH, HIF, WT1, PARP inhibitors, hypomethylating agents, decitabine, and azacytidine (Figure 1). Due to the large numbers of articles published on the therapeutic potential of vitamin C in cancer, we apologize to those authors whose work was not cited in this review.

## 3. Chemical Forms of Vitamin C

**Figure 2:**
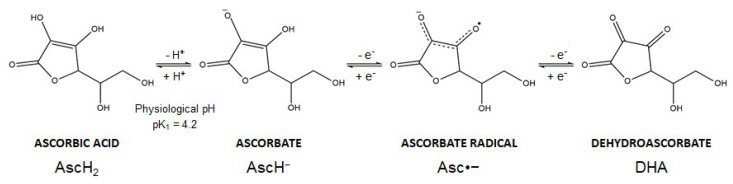
Vitamin C has different chemical structures. At physiological pH, ascorbic acid loses a proton to form ascorbate, which can donate two electrons sequentially. Loss of the first electron (oxidation) generates ascorbate radical and the loss of the second electron produces dehydroascorbate. See text for further details.

Vitamin C is the common name of L-ascorbic acid, which can be found in different chemical forms. Ascorbic acid (AscH_2_) (a Latin-derived word, meaning “without scurvy”) is highly soluble in water, but the presence of two ionizable hydroxyl groups makes the compound pH sensitive (pK_1_ = 4.2; pK_2_ = 11.6). In biological systems, ascorbic acid loses a proton forming the ascorbate anion (AscH^−^), the reduced dominant form of vitamin C at physiological pH. Thereafter, AscH^−^ undergoes oxidation and this reaction is dependent on pH and is accelerated by catalytic metals (e.g., iron). In particular, AscH^−^ undergoes one-electron oxidation to form the ascorbate radical (Asc^•−^). Donation of the second electron gives rise to dehydroascorbic acid (DHA), the fully oxidized form of vitamin C (Figure 2). The ascorbate radical is relatively unreactive since two ascorbate radical anions (Asc^•−^) can form a dimer and further undergo a disproportionation reaction to form DHA and AscH^−^ [16,17]. These oxidation reactions are coupled with reactive oxygen species (ROS) formation and metals reduction. Spontaneous autoxidation may also occur, but at pH 7 this reaction is very slow.

After transporter-mediated entry into the cell, the DHA form is reduced back to ascorbate. This reaction involves glutathione (GSH) and enzymatic activities like glutaredoxin or other dehydroascorbate reductases with generation of the oxidized glutathione disulfide form (GSSG). The subsequent reduction in GSSG is mediated by the NADPH-dependent glutathione reductase [18]. Thus, cells like erythrocytes or astrocytes may efficiently recycle extracellular DHA by taking it up through GLUT transporters (see also below) [19]. This process allows the recycling and reuse of ascorbate for cellular processes, allowing the antioxidant activity of vitamin C to occur.

Ascorbate also donates electrons to metals such as copper and iron, regulating the activity of enzymes belonging to the families of copper-containing monooxygenases and Fe^2+^-dependent and α-ketoglutarate-dependent dioxygenases (αKGDD). The first family includes the dopamine β-monooxygenase, necessary for the synthesis of norepinephrine, and the peptidylglycine α-amidating monooxygenase, responsible for a post-translational modification required for the full activation and stabilization of bioactive peptides (e.g., neuroendocrine peptides) [20]. The second and larger family includes several hydroxylases involved in various functions such as type 1 collagen synthesis, carnitine synthesis, tyrosine catabolism, stability of hypoxia-inducible factor α (HIF-1α) and epigenetic modifications [20,21,22,23]. The ability to donate one or two electrons makes ascorbate an excellent reducing agent and antioxidant system in humans [20,24,25,26].

## 4. Vitamin C Transport into the Cells

In humans, vitamin C is obtained from the diet that contains both ascorbate and DHA. Ascorbate entry into cells is mediated by sodium-dependent vitamin C transporters (SVCT), which comprise two isoforms SVCT1 and SVCT2 (encoded by the SoLute Carrier family 23 member 1 and 2 genes, *SLC23A1* and *SLC23A2*, respectively) that actively co-transport sodium and ascorbate [17,27]. These transporters bind first one molecule of Na^+^, then one molecule of ascorbate, and finally an additional Na^+^ [28]. Ascorbate entry into tissues from blood vessels is mediated by a para-cellular movement of ascorbate through gaps between endothelial cells, even though these cells express a large number of SVCT2 [27,29]. SVCT1 is expressed at the apical level in the epithelial intestinal and renal proximal tubules cells in addition to liver and lung [30,31,32]. SVCT1 is involved in the intestinal absorption of vitamin C and in its renal re-absorption back to the blood [33]. On the other hand, SVCT2 is expressed throughout the body tissues (with the exception of red blood cells), including the basolateral side of intestinal epithelial cells [17,31,34,35,36]. The uptake of ascorbate is very tightly controlled. In fact, these transporters are sensitive to intracellular ascorbate concentrations, being upregulated or downregulated in the presence of low or high ascorbate levels, respectively. Therefore, this uptake-pathway maintains the homeostatic physiological concentration of vitamin C in the blood [37,38]. When Ca^2+^ and Mg^2+^ are absent, the SVCT2 transport system is in an inactive conformation, despite the presence of Na^+^ [28].

Another possibility for vitamin C entry into the cells is in its DHA form that is transported through a facilitated diffusion mechanism by glucose transporters (GLUT) that belong to the SLC2 family. GLUT2 and GLUT8 (encoded by the *SLC2A2* and *SLC2A8* genes, respectively) are expressed only in the intestine and are used by DHA to enter into the enterocytes [39]. Although, a diet rich in free sugars inhibits DHA gut absorption, complex carbohydrates do not affect DHA transport due to the glucose release in jejunum [39]. In this way, DHA can be absorbed in the duodenum by GLUT2 and GLUT8 [39]. In all other human tissues, DHA competes with glucose to be transported via GLUT1 and GLUT3 isoforms (encoded by the *SLC2A1* and *SLC2A3* genes, respectively) [40].

The GLUTs affinity for DHA (Km ~1–3 mM) is lower than SVCTs affinity for ascorbate (Km ~20–100 μM) [17,36,41,42]. In normal conditions, the glucose concentration (2–5 mM) in the blood is much higher than DHA (˂2 μM); therefore, cell uptake of ascorbate through SVCT2 is preferred [17,30]. Moreover, cells may change their transporter expression depending on vitamin C plasma and intracellular concentrations [17]. Glucose blood concentration and receptor affinity markedly influence the uptake of DHA. Interestingly, the rate of DHA uptake via GLUT1 and GLUT3 in cancer cells is faster than the uptake of ascorbate through SVCT2, even in the presence of glucose [27,43]. In tumor microenvironment oxidizing conditions, the prominent extracellular form of vitamin C is likely DHA that is taken up by the cells and rapidly reduced back to ascorbate, creating a steep gradient across the cell membrane. Furthermore, due to the high requirement of glucose by cancer cells for their metabolism, the GLUT transporters are up-regulated, contributing to DHA intake [44,45,46,47].

## 5. Vitamin C Dose-Dependent Pharmacokinetics

The main factors that affect the bioavailability of vitamin C are the absorption rate at the intestinal level (in the case of oral formulations or dietary intake) and renal re-absorption. Vitamin C plasma levels show a dose-dependent pharmacokinetics and a first order kinetics of elimination [48,49,50,51]. Studies in humans have revealed that oral doses exceeding 250 mg/day produce plateau plasma concentrations that never exceed 100 µM [4,52]. Phase 1 studies identified 3 g of vitamin C orally administered every 4 h (12 g/day) as the maximum tolerated dose, with maximum plasma concentrations of 220 µM [53]. When plasma ascorbate levels are lower than physiological plasma concentrations (oral doses 0.1 g/d) (i.e., in deficient intake periods), the kidney actively re-absorbs ascorbate back into the bloodstream, preventing the occurrence of acute scurvy [52]. In this condition, the half-life of plasma ascorbate is long (days). In contrast, when the plasma ascorbate levels are higher than 70–80 µM (oral doses >100 g/d), renal excretion increases because of saturated tubular re-absorption at the kidney level and the ascorbate plasma half-life is very short (~30 min) [48,54,55,56].

On the contrary, studies performed in cancer patient have demonstrated that after I.V. administration, the tight control mechanisms of intestinal absorption are by-passed and the observed vitamin C plasma concentrations are in the millimolar range (around 100-fold higher than those detected after oral doses). In particular, vitamin C reaches a plasma peak higher than 20 mM and shows a half-life of 2 h (1.7 h–2.5 h) [50,57]. Moreover, the elevated inflammation and oxidative stress presents in cancer patients results in increased vitamin C utilization and lower plasma levels comparing to healthy people [58,59,60]. Overall, since vitamin C pharmacokinetic properties depend on the route of administration used, the results of studies with oral or intravenous doses are not directly comparable [53]. Table 1 indicates the different plasma concentrations obtained after oral or I.V. administration of different doses of vitamin C. 

## 6. Mechanisms of Vitamin C Anticancer Action

The antitumor effects of vitamin C have been consistently demonstrated by using in vitro cultures of cancer cell lines of different tissue origin (e.g., ovarian, pancreatic adenocarcinoma, lymphoma) and in vivo murine models [65,66,67,68,69,70,71,72,73,74].

### 6.1. Vitamin C as Pro-Oxidant Agent

**Figure 3:**
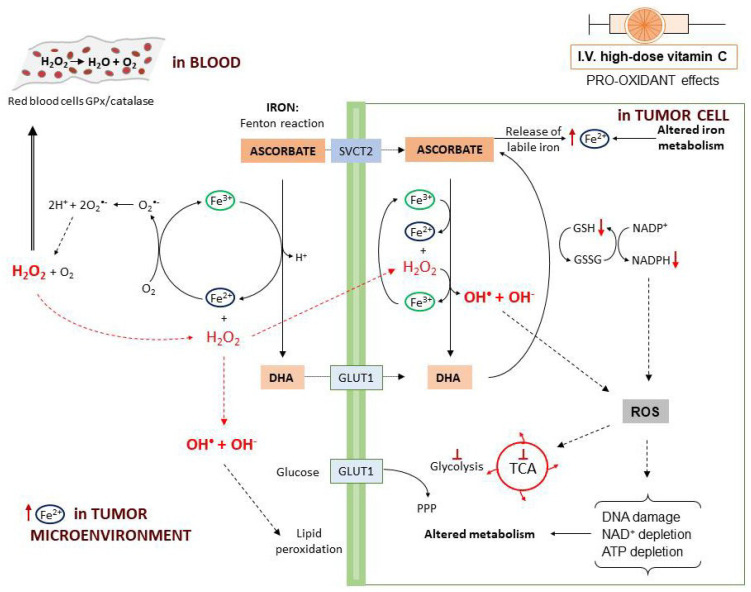
Pro-oxidant effects of intravenously administered high-dose vitamin C. Intravenous administration (I.V.) of high-dose vitamin C (plasma ascorbate levels >1 mM) induces cytotoxic effects against cancer cells through different mechanisms of action. Ascorbate reacts with Fe^3+^ via Fenton reaction leading to the formation of Fe^2+^ that, by reacting with H_2_O_2_, produces highly damaging hydroxyl radicals. In the blood, H_2_O_2_ is eliminated by ROS scavenger systems [e.g., the glutathione peroxidase (GPx) and catalase] present on the erythrocyte membrane. In the extracellular matrix of the tumor microenvironment, hydroxyl radical accumulation can induce direct damage to cell membrane by lipid peroxidation; H_2_O_2_ can also enter into the cell by diffusion. Vitamin C enters into the cell by using different transporters: (1) via SVCT2 as ascorbate; (2) via GLUTs as DHA produced by ascorbate oxidation. Inside the cells, DHA is converted back to ascorbate decreasing GSH activity and NADPH. Tumor cells contain higher levels of labile iron (Fe^2+^) due to altered iron metabolism and high intracellular concentrations of ascorbate may favor the release of Fe^2+^ from ferritin. Tumors may also secrete in the extracellular matrix high amounts of ferritin from which Fe^2+^ can be released either directly by ascorbate or indirectly through the production of O_2._ ROS accumulation induces ATP depletion and causes cancer cell death as a consequence of DNA damage, GAPDH inhibition and NAD+ depletion, metabolism alteration due to glycolysis blockade, tricarboxylic acid cycle (TCA) disruption and shift toward the pentose phosphate pathway (PPP).

The first mechanism described to explain vitamin C antitumor activity relies on the pro-oxidant effects observed following high-dose administration. The pro-oxidant properties have been attributed to the ability of ascorbate to reduce Fe^3+^ to Fe^2+^ with consequent generation of ROS through the Fenton reaction (Figure 3). In fact, tumor cells contain higher levels of labile iron (Fe^2+^) compared to normal cells and this favors higher ROS generation [75]. Moreover, when administered at high dosage, ascorbate may induce the release of Fe^2+^ from storage proteins [76]. Vitamin C-induced ROS production is further potentiated by the presence of O_2_ [77].

In particular, a study on human Burkitt’s lymphoma cells reported that, at 2 mM concentration, vitamin C is oxidized to the ascorbate radical with production of H_2_O_2_ [65]. In a rat model, it was found that parenteral administration of vitamin C induced H_2_O_2_ production depending on the dose. Furthermore, high-dose ascorbate may induce H_2_O_2_ formation through induction of members of the NADPH oxidase family (DUOX1 and 2) [78]. When plasma ascorbate levels are higher than 1 mM, in the interstitial (extracellular) fluids the ascorbate radical concentration exceeds 100 nM [66,67]. In the blood, H_2_O_2_ is rapidly reduced back to H_2_O by the glutathione peroxidase (GPx) and catalase reduction systems on the erythrocytes plasma membrane. In this way, H_2_O_2_ is undetectable in the blood [65,66]. On the contrary, in the extracellular matrix, H_2_O_2_ undergoes accumulation and, in the tumor microenvironment, by interaction with Fe^2+^ can generate hydroxyl radicals that induce cell damage externally, through membrane lipid peroxidation [65,66]. After entering into the cells, H_2_O_2_ reacts with intracellular Fe^2+^ leading to a continuous production of highly damaging hydroxyl radicals. This ROS accumulation directly damages mitochondria and DNA with consequent poly(ADP-ribose) polymerase (PARP) overactivation and NAD+ depletion [79]. In the presence of DHA, the consumption of NADPH used to generate GSH from GSSG eventually results in blockade of glycolysis [46,80]. Ascorbate also induces metabolic shift toward pentose phosphate pathway (PPP), glycerol synthesis and disruption of the tricarboxylic acid cycle (TCA) [81]. Moreover, ROS production leads to inhibition of glyceraldehyde 3-phosphate dehydrogenase (GAPDH) and this contributes to glycolytic blockage. In a *KRAS/BRAF* mutated colorectal cancer cell model, a reversible oxidation of GAPDH was observed after vitamin C treatment [46]. On the other hand, in a neuroblastoma cell line, vitamin C-induced cell death was prevented by the addition of NAD+, demonstrating that GAPDH was inhibited as a consequence of NAD+ depletion [73]. These effects cumulatively lead to ATP depletion and cell death [46,68,81,82,83].

Depending on their levels, ROS have been shown to exert both beneficial and deleterious effects [84]. It is recognized that, compared to normal cells, cancer cells have a greater amount of basal intracellular ROS that promote tumor progression [85,86,87,88]. Tumor cells can tolerate elevated ROS levels, which derive, at least in part, from the glucose-dependent cancer cell metabolism (Warburg effect), by increasing the expression of GSH system. High-dose vitamin C may kill tumor cells either because it alters important cell signaling pathways regulated by ROS (e.g., cell proliferation, migration, neovessel formation) or because it further increases ROS levels causing cellular damage beyond the available defenses systems [89]. Moreover, vitamin C may decrease the antioxidant cellular defenses by selectively reducing the GSH content in tumor cells and not in normal cells [90,91,92]. These aspects have been thoroughly discussed in a recently published review [18].

### 6.2. Vitamin C as Enzymatic Regulator of TET Enzymes

Vitamin C has recently been found to be an epigenetic modulator through its effects on Ten Eleven Translocation (TET) enzymes, a family of αKGDD enzymes involved in active DNA demethylation (direct removal of a methyl group independently of DNA replication) [93,94]. TET enzymes catalyze the oxidation of 5-methylcytosine (5mC) to 5-hydroxymethylcytosine (5hmC), 5-carboxylcytosine (5fC) and 5-formylcytosine (5cC), followed by conversion to cytosine by the base excision repair (BER). An altered regulation of these enzymes is implicated in tumor development and maintenance. *TET* mutations (mostly *TET2*) result in nonfunctional forms of the enzyme, leading to gene promoter hypermethylation. Vitamin C acts as a co-factor for TET enzymes through a direct interaction with their C-terminal catalytic domain and, to a lesser extent, by reducing Fe^3+^ to Fe^2+^, making the latter available for TET activity [94,95]. TET function and intracellular vitamin C are both involved in reprogramming and maintaining self-renewal of stem cells [96,97,98].

**Figure 4:**
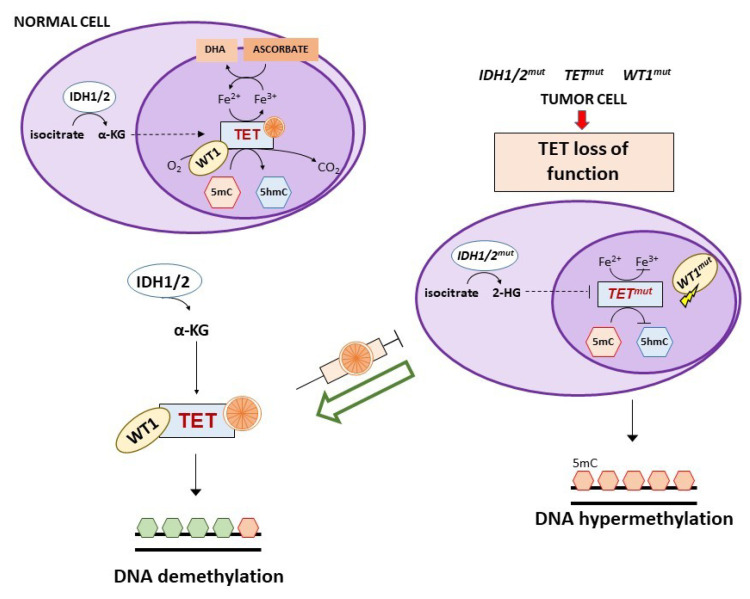
Activity of high-dose vitamin C in tumors with TETs, IDH1/2 or WT1 altered pathways. TETs are a family of αKGDD enzymes involved in active DNA demethylation that catalyze the oxidation of 5-methylcytosine (5mC) to 5-hydroxymethylcytosine (5hmC). *TET* mutations (mostly *TET2*) result in nonfunctional forms of the enzyme leading to hypermethylation gene promoters. IDH1/2 enzymes catalyze the oxidative decarboxylation of isocitrate to α-ketoglutarate (αKG) that is required for the activity of multiple dioxygenases, including TETs. Gain-of-function mutations of *IDH1/2* result in the overproduction of the oncometabolite 2-hydroxyglutarate (2-HG) that inhibits TET activity. WT1 interacts with TET2 and recruits it to the promoter of WT1-target genes stimulating their demethylation and expression. WT1 mutations hamper the ability of TET2 to bind to and induce transcriptional activation of WT1-target genes. High-dose vitamin C mimics TET demethylating activity and restores the normal DNA methylation pattern inhibiting tumor progression. See the text for further details.

The three TET1, TET2, and TET3 members have a different tissue distribution and appear to be altered in certain tumors. In particular, *TET2* is frequently mutated in both myeloid and lymphoid hematological malignancies and restoration of TET2 blocks aberrant self-renewal of pre-leukemic stem cells [99,100,101,102]. Consistently, in acute myeloid leukemia (AML) cells with *TET2* mutations, treatment with vitamin C mimicked TET2 restoration by increasing TET activity (Figure 4) and blocked leukemia progression in patient-derived tumor xenograft models [102]. However, the ability of vitamin C to restore TET2 activity seems to depend on N- and C-terminal lysine acetylation and type of TET2 mutations [103].

*TET2* mutations are mutually exclusive to isocitrate dehydrogenase (*IDH*) *1/2* or Wilms tumor protein 1 (*WT1*) mutations that can also be detected in AML and myelodysplastic syndromes [104]. IDH1/2 enzymes catalyze the oxidative decarboxylation of isocitrate to α-ketoglutarate (αKG) that is required for the activity of multiple dioxygenases, including TETs. Gain-of-function mutations of *IDH1/2* result in the overproduction of the oncometabolite 2-hydroxyglutarate (2-HG) that is able to inhibit TET2 through a competitive mechanism (Figure 4) [105,106,107]. In *IDH1* mutant mouse bone marrow cells, vitamin C (added daily at 100 μg/mL corresponding to 0.325 mM, in the form of 2-phosphate L-ascorbic acid) was found to overcome the effects of *IDH1* mutations, promoting DNA demethylation and epigenetic remodeling of transcription factor-binding sites through stimulation of TET2 activity with consequent induction of leukemia cell differentiation [108]. Of interest, 2-phosphate L-ascorbic acid is a compound that is stable in cell culture and does not induce the production of extracellular H_2_O_2_, allowing to study only the activity of vitamin C as enzymatic regulator [108,109]. *IDH1/2* mutations are also detected in solid tumors (e.g., glioma, colorectal, breast, renal cancers) and in an *IDH1* mutated colorectal cancer cell line, treatment with vitamin C synergized with an IHD1 inhibitor by rescuing TET activity [110].

Prolonged exposure to vitamin C, at concentrations capable of regulating αKGDDs enzymatic activity, may induce epigenomic remodeling. In blast cells of leukemia patients with *TET2* mutations, aberrant promoter methylation and reduction in 5hmC at the level of gene enhancers were detected [111]. In human kidney cancer cells lines, restoration of DNA 5hmC levels after protracted vitamin C exposure was also observed [112]. Furthermore, in *IDH1* mutant mouse bone marrow cells, vitamin C induced differentiation and maturation of myeloid progenitor cells [108]. Through this mechanism, vitamin C may counteract the epigenetic dysregulation associated with cancer development and progression, which leads to aberrant gene expression and genomic instability.

Pharmacological doses of vitamin C were reported to reduce DNA methylation and to restore 5hmC DNA levels via TET2 activity even in tumors with functional loss of TET2 unrelated to gene mutations or transcriptional inactivation. Recently, low levels of 5hmC have been proposed as an independent adverse prognostic marker in tumors such as cutaneous T-cell lymphoma and clear cell renal cell carcinoma [113,114,115]. The latter tumor shows DNA cytosine hypermethylation, especially at the level of tumor-suppressor genes, that has been attributed to low expression of L-2-hydroxyglutarate dehydrogenase (L2HGDH) with consequent overproduction of the 2HG oncometabolite (the L isoform) that in turn causes functional inactivation of TET2. Treatment with vitamin C reduced DNA methylation and restored 5hmC levels via TET activation and inhibited tumor growth in vitro and in vivo [115].

WT1 is a transcription factor that regulates many cellular pathways, including WNT and MAPK signaling, and is involved in processes like cell differentiation and tumor suppression. This transcription factor interacts with TET2 and recruits it to the promoter of genes regulated by WT1 favoring their demethylation and expression [104,116]. Mutations of *WT1* hamper the ability of TET2 to bind to and induce transcriptional activation of WT1-target genes (Figure 4). A clinical study on *WT1*-mutated AML refractory to induction chemotherapy suggested the use of vitamin C as adjunct therapy, based on the evidence that *WT1* mutant leukemia cells show low 5hmC levels that in turn indicate reduced TET2 activity [15]. Since *WT1* mutations are present in a large number of tumors, it is likely that vitamin C treatment might be useful also in other clinical settings besides AML [104].

### 6.3. Vitamin C as Enzymatic Regulator of Others αKGDDs

HIF hydroxylases are another class of αKGDDs enzymes that are affected by vitamin C. In human melanoma cell lines, vitamin C was found to act as cofactor of HIF hydroxylases, which induces recognition of HIF-1α by the von Hippel–Lindau tumor suppressor protein (VHL) with consequent ubiquitination and proteasomal degradation [117]. The heterodimeric transcription factor HIF1 comprises a cytosolic, O_2_ sensitive subunit (HIF-1α) and a constitutively expressed subunit (HIF-1β). Under oxygen deficiency conditions (e.g., ischemia, tumors), HIF-1α undergoes hydroxylation on specific proline and asparagine residues and proteasomal degradation is prevented. In this way, HIF-1α can translocate to the nucleus, dimerize with HIF-1β and activate target genes involved in the regulation of many cellular functions such as proliferation, apoptosis, cell migration, angiogenesis, glucose transport, and metabolism [118]. In various tumors, HIF-1α is constitutively activated and high levels of expression seem to correlate with vitamin C cytotoxicity [119,120,121]. Moreover, loss of function mutations in succinate dehydrogenase and fumarate dehydrogenase can increase succinate and fumarate levels, which, in turn, may lead to competitive inhibition of HIF hydroxylases and HIF-1α constitutive activation [18,119]. In several human cancers (endometrial, colorectal, breast, and thyroid cancer), an inverse correlation between HIF-1α and intracellular vitamin C levels was also found [122,123,124]. In AML patients, high expression levels of HIF-1α and GLUT1 were associated with lack of response to chemotherapy, probably due to the higher glycolytic metabolism of resistant tumor cells [125]. Moreover, high HIF-1α activity has been reported to inhibit TET2 expression [126]. Therefore, in tumors with HIF1 overexpression or overactivation, vitamin C treatment may increase the activity of HIF hydroxylases, with consequent HIF1α degradation and inhibition of the tumor promoting effects of this transcription factor [18,127,128,129,130]. However, in patients with clear-cell renal cell carcinomas with mutations in the *VHL* gene that prevent the degradation of hydroxylated HIF-α with consequent accumulation of the transcription factor, there was no association between HIF activity and ascorbate content [131]. These data suggest that vitamin C treatment is unlikely to be effective in VHL-defective tumors. Conversely, in VHL-proficient tumors with increased HIF activity due to hypoxic conditions, high-dose vitamin C might prove beneficial by enhancing HIF-α degradation through stimulation of HIF hydroxylase enzyme activity [131]. Nevertheless, since HIF positively regulates GLUT1 expression, this might favor the entry of DHA into VHL-defective cells sensitizing them to vitamin C cytotoxic effects [119,132]. Interestingly, administration of ascorbate by intravenous infusion to colon cancer patients resulted in increased ascorbate content within the tumor and reduced expression of HIF-dependent proteins [133].

Other epigenetic regulators belonging to the αKGDDs class are the Jumonji C-domain-containing histone demethylases (JHDM). These enzymes catalyze the histone demethylation at arginine and lysine residues regulating chromatin-dependent processes. Some evidence demonstrated that vitamin C may modulate JHDM activity affecting their role in embryonic stem cell reprogramming [134]. However, further studies are required to clarify the ability of vitamin C to modulate JHDM activity in cancer [40].

### 6.4. Vitamin C may Favor DNA Damage by Increasing 5-Hydroxymethylcytosine Levels

DNA damage induced by vitamin C can occur at in vitro pro-oxidant concentrations (>1 mM), as well as at concentrations capable of regulating αKGDDs enzymatic activity (0.25–1 mM) [102,108,112,135,136]. In the latter case, vitamin C-induced DNA damage derives from its ability to increase 5hmC levels.

In recent years, several studies have demonstrated a fundamental role of 5hmC as the most stable oxidized 5mC intermediate in the DNA demethylation process [137]. As described above, low 5hmC levels were reported as marker of TET dysregulation and vitamin C was found to enhance TET activity mimicking the action of hypomethylating agents [94,102,114,135,138,139,140,141,142,143,144,145,146,147]. In tumor tissues, lower 5hmC levels are generally detected compared to normal tissues that have been attributed to down-regulation of TET activity by tumor hypoxic conditions since O_2_ is required for TET function [148,149]. Other pathways that can explain the low amount of 5hmC in tumor DNA include: (a) passive dilution by cell division in the presence of defective activity of DNA methyltransferase 1 (DNMT1); (b) changes in TET activity as a consequence of gene mutations (see Section 6.2) or TET protein “de-localization” [150,151].

Vitamin C is known to induce single strand breaks and to activate the BER pathway via TET-mediated DNA oxidation [102,152]. Moreover, the presence of 5hmC at stalled replication forks acts as a recruitment marker for the BER component apurinic and apyrimidinic endonuclease 1 (APE1) [153]. In a BRCA2-deficient murine cellular model, Kharat and colleagues demonstrated that low levels of 5hmC and TET2 expression were associated with increased stability of stalled replication forks and resistance to PARP inhibitors [153]. PARP inhibitors are a class of anticancer drugs approved for the treatment of tumors with defective DNA repair due to germline and somatic mutations or epigenetic alterations of essential components (e.g., BRCA1 or BRCA2) of the homologous recombination system that is involved in the repair of DNA double strand breaks [154]. PARP inhibitor-induced trapping of PARP1 at DNA damage site impairs the progression of the replication fork and the repair/restarting of stalled replication fork requires a fully active homologous recombination. In homologous recombination-deficient cells, the intervention of error-prone DNA repair processes (e.g., non-homologous end-joining) can induce genomic instability and rearrangements, which eventually lead to tumor cell death and synthetic lethality [155,156]. Moreover, PARP1 trapping triggers excessive fork degradation of stalled replication forks resulting in fork collapse and DNA double strand breaks [157]. On this basis, in BRCA-deficient tumors, protection of stalled replication forks may contribute to the development of resistance to PARP inhibitors. In the presence of TET2 functional defects and consequent low 5hmC levels, the recruitment of APE1 on the stalled replication fork is impaired and this impedes the degradation of stalled replication forks [153]. In this context, exposure to vitamin C was found to increase 5hmC via TET2 activity, by restoring the recruitment of APE1 on stalled replication forks and inducing their degradation [153,158]. However, additional studies are required to establish whether vitamin C, by increasing 5hmC levels, might contribute to restore the sensitivity of tumors resistant to PARPi or to other chemotherapeutic agents that induce a DNA damage response involving the homologous recombination system (e.g., cisplatin).

## 7. High-Dose Vitamin C Exerts Preferential Cytotoxic Activity against Cancer Cells

What makes vitamin C interesting as antitumor agent is that it selectively kills cancer cells, without harming normal cells [46,65,67,68,91,159,160]. This is confirmed by the results of clinical studies indicating that high-doses of vitamin C are generally well tolerated [57,60,62,64,133,161].

Mechanisms involved in the preferential sensitivity to tumor cells to high-dose vitamin C, include high levels of Fe^2+^ in the tumor microenvironment as well as intracellularly, with consequent higher production of H_2_O_2_ and damaging hydroxyl radicals compared to normal cells. Although H_2_O_2_ is regarded as a major ROS involved in vitamin C pro-oxidant activity, cell damage is mainly mediated by hydroxyl radicals that are generated via Fenton reaction through the interaction of Fe^2+^ with H_2_O_2_ [84,162]. Cancer cells may show alterations in iron metabolism due to up-regulation of transferrin receptor or down-regulation of iron export (ferroportin 1) with consequent increase in iron intracellularly [163]. Moreover, certain tumors secrete in the extracellular matrix high amounts of ferritin from which Fe^2+^ can be released either directly by ascorbate or indirectly through the production of O_2_^•−^ (Figure 3) [164]. Extracellular H_2_O_2_ may also favor the oxidation of ascorbate to DHA that is then rapidly transported into tumor cells expressing high levels of GLUT1, generating oxidative stress [18]. To this regard, vitamin C has been recently shown to increase GLUT1 expression in cancer cells and to induce the opposite effects in normal cells [165]. Finally, specific molecular defects present in cancer cells may render them more vulnerable to vitamin C-mediated antitumor effects (see Section 6.2).

In the majority of cases, patients with hematological malignances or solid tumors such as melanoma, lung, head and neck squamous carcinoma, hepatocellular carcinoma, and breast cancer, have lower plasma levels of vitamin C compared to healthy people [58,166,167,168,169,170,171,172,173,174,175,176]. More complex is the quantification of vitamin C inside the cells. Vitamin C cell content in the tissues of healthy subjects (blood, neurons, glial cells; 1–10 mM range) exceeds the plasma concentration (equivalent to 0.04–0.08 mM) except in the case of erythrocytes, in which vitamin C intracellular concentration corresponds to that detected in plasma [4,17,52,177]. Particularly high is the concentration of vitamin C in lymphocytes, monocytes, and platelets, which are considered reservoirs of vitamin C. On the other hand, high-grade endometrial tumor tissues, colorectal, and breast cancer showed lower levels of vitamin C compared to lower grade tumors or adjacent normal tissues [119,122,123]. In the latter case, ascorbate tumor content directly correlated with disease-free survival [122]. In breast cancer, the vitamin C content was instead higher than in the non-neoplastic surrounding tissue [178,179]. However, to our knowledge, limited information is currently available on vitamin C levels present in tumors vs. normal tissues and data are not always easily comparable.

The different vitamin C content reported in the tumor vs. normal cells might depend on the expression pattern of transporters. Indeed, in several tumors (e.g., *KRAS/BRAF* mutated colorectal, gastric, or breast cancer) the sensitivity to vitamin C correlated with the expression of GLUT1 and DHA adsorption [44,45,46,47,180,181]. Interestingly, the activity of vitamin C against *KRAS/BRAF* mutated colorectal cancer might be useful to counteract tumor resistance to anti-epidermal growth factor receptor (EGFR) monoclonal antibodies (i.e., cetuximab, panitumumab) [182].

Liu and collaborators recently showed a lower expression of the *SLC2A3* gene (GLUT3), but not of the *SLC2A1* gene (GLUT1), in leukemia blasts compared with normal hematopoietic cells [183]. Moreover, exposure to vitamin C of the OCI-AML3 cell line, which does not express GLUT3, did not result in increased cytoplasmic levels of the vitamin and did not affect leukemia cell survival [183]. Additionally, in breast cancer cells, SVCT2 expression led to a higher uptake of vitamin C, increased ROS production and cytotoxicity [184]. Furthermore, the cytotoxic effects exerted by ascorbate in acute and chronic myeloid leukemia cell lines are dose-dependent and already evident at in vitro concentrations of 250 µM that do not induce significant production of H_2_O_2_ [102,159,183].

Concerning normal cells, neutrophils have been reported to accumulate vitamin C via SVCT2, showing intracellular levels of 1–2 mM [185]. Interestingly, neutrophils isolated from healthy individuals did not show additional uptake of ascorbate likely due to saturation of its transporters (0.35 nmol/10^6^ cells), but they were able to rapidly absorb DHA that, however, did not induce any cytotoxic effect [186,187]. Moreover, single nucleotide polymorphisms of *SLC23As* (SVCT) genes, identified in several tumors (e.g., gastric cancer) may affect vitamin C plasma concentration and intracellular content [188,189,190].

Overall, these studies indicate that substantial differences may exist among the various cell histotypes in terms of receptors and absorption rate of vitamin C. Therefore, future studies aimed at understanding the preferential uptake of vitamin C by cancer cells should not only include analysis of the expression of GLUT1, GLUT3, and SVCT2 transporters but also measurement of vitamin C content inside the cells after treatment.

## 8. Conclusions

Most of the reported cytotoxic effects induced by vitamin C are observed at concentrations that can only be reached after I.V. administration of high doses. However, due to the short half-life detected after I.V. injection, these concentrations cannot be maintained for a prolonged time. Indeed, weekly I.V. administration of high-dose vitamin C to prostate cancer patients did not induce tumor regression [50,64]. Thus, it has been suggested that in patients with an intact kidney function, a bolus loading dose followed by a maintenance continuous infusion is likely required to achieve a steady-state plasma concentration of vitamin C in the millimolar range [50].

In clinical trials, intravenous vitamin C is generally tested as adjunct treatment to chemotherapy, since vitamin C as monotherapy often failed to meet efficacy endpoints [18,60,62,64,191,192]. However, it should be noted that many of the first studies testing vitamin C as single agent were done in terminal patients without tumors stratification and a clear definition of efficacy endpoints [10,11,193]. Based on the results of preclinical studies, future randomized clinical trials should be designed to evaluate vitamin C activity in specific tumor molecular subtypes, such as those characterized by HIF-1α over-expression and TET2, L2HGDH, IDH1/2, or WT1 altered pathways [194]. To this regard, a recent study has demonstrated that intravenous treatment with ascorbate induced clinical remission in a patient with AML carrying TET2 and WT1 mutations in separate clones, after failure of induction chemotherapy [15].

For combinations therapies, clinical studies should be planned taking into account the different molecular mechanisms involved in vitamin C antitumor activity that can be observed even at lower concentrations, such as induction of DNA demethylation [167,195,196]. In particular, the conversion of 5mC to 5hmC deriving from vitamin C-mediated activation of TETs may synergize with passive DNA demethylation (i.e., absence of methylation of newly synthesized DNA strands) obtained by azacytidine or decitabine that are inhibitors of DNMT1 [197,198,199]. Indeed, I.V. administration of low-dose vitamin C in combination with decitabine to elderly AML patients resulted in enhanced TET2 activity and improved clinical response [200].

As described above, vitamin C might have a role in modulating the BER pathway and has been shown to enhance the activity of PARP inhibitors reducing the viability of human AML and promyelocytic leukemia cell lines [102,201]. Indeed, a case study on eight cancer patients with advanced stage malignancies characterized by defects in the homologous recombination repair system showed that I.V. vitamin C in combination with PARP inhibitors (niraparib or olaparib or talazoparib) induced 37.5% complete remissions (3 patients out of 8) and 62.5% (5 patients out 8) partial responses [202]. Despite the positive results, further clinical studies on a larger number of patients are required to better clarify the clinical efficacy of vitamin C in combination with these agents.

Overall, intravenously administered high-dose vitamin C is generally safe [203,204]; however, in some patients severe adverse effects may be observed. In particular, toxicity includes oxalate nephropathy in patients with renal impairment due to increased urinary excretion of oxalate as end-product of vitamin C metabolism, severe hemolysis in patients with paroxysmal nocturnal hemoglobinuria or with glucose-6-phosphate dehydrogenase deficiency due to erythrocyte inability to maintain glutathione in its reduced form, increased risk of thrombosis in cancer patients due to procoagulant activation of erythrocytes [205,206]. Thus, vitamin C treatment should also take into account patient-specific risk factors for these adverse effects.

## Figures and Tables

**Table 1:** Vitamin C plasma concentrations under different conditions and route of administrations.

Vitamin C Dose	Plasma Concentration	References
	μM	
0.075–0.125 g/d Normal intake ~0.030 g/d Depletion 0.010 g/d Deficiency	40–80 11.4–28.4 <11.4	[4,5,6,7,53,61]
*Oral route*		
Dietary intake 0.2–0.3 g/d (fruits and vegetables)	70–85	[53]
Single dose 0.2–1.25–3 g/d	90–157–206	[53]
Maximum tolerated dose 18 g/d (3 g every 4 h)	≤220	[53]
Oral daily dose:		[4]
0.03 g	8.7 ± 0.8	
0.1 g	55.9 ± 3.6	
0.2 g	65.7 ± 3.8	
0.4 g	70.0 ± 4.4	
*I.V. route*	mM	
1.25 g	0.9 ± 0.2	[4,53]
3 g	1.8	
5 g	2.9	
10 g	5.6	
50 g	13.4	
100 g	15.4	
30 g/m^2^	23 ± 9	[57]
70 g/m^2^	49 ± 8	
90 g/m^2^	49 ± 14	
0.1 g/kg (~3.7 g/m^2^)	2.4 ± 0.3	[62]
0.2 g/kg (~7.4 g/m^2^)	4.7 ± 0.5	
0.4 g/kg (~14.8 g/m^2^)	8.5 ± 0.6	
0.6 g/kg (~22.2 g/m^2^)	11.3 ± 2.4	
0.9 g/kg (~33.3 g/m^2^)	17.0 ± 3.6	
1.5 g/kg (~55.5 g/m^2^)	26.2 ± 4.9	
*I.V. infusion (grams/wk) plus oral dose (0.5 g/d)*		
5 g week 1	1.9 ± 0.4	[50,63,64]
30 g week 2	12.6 ± 3.4	
60 g week 3	19.6 ± 6.8	
60 g week 4	21.1 ± 5.0	
